# Exposure to Bisphenol A and Other Phenols in Neonatal Intensive Care Unit Premature Infants

**DOI:** 10.1289/ehp.0800265

**Published:** 2008-12-10

**Authors:** Antonia M. Calafat, Jennifer Weuve, Xiaoyun Ye, Lily T. Jia, Howard Hu, Steven Ringer, Ken Huttner, Russ Hauser

**Affiliations:** 1 Centers for Disease Control and Prevention, Atlanta, Georgia, USA;; 2 Rush Institute for Healthy Aging, Rush University Medical Center, Chicago, Illinois, USA;; 3 Department of Environmental Health, Harvard School of Public Health, Boston, Massachusetts, USA;; 4 Schools of Public Health and Medicine, University of Michigan, Ann Arbor, Michigan, USA;; 5 Brigham and Women’s Hospital, Harvard Medical School, Boston, Massachusetts, USA;; 6 Neonatology Unit and; 7 Vincent Memorial Obstetrics and Gynecology Service, Massachusetts General Hospital, Harvard Medical School, Boston, Massachusetts, USA

**Keywords:** benzophenone, biomonitoring, BPA, glucuronidation, neonate, NICU, paraben, triclosan

## Abstract

**Objective:**

We previously demonstrated that exposure to polyvinyl chloride plastic medical devices containing di(2-ethylhexyl) phthalate (DEHP) was associated with higher urinary concentrations of several DEHP metabolites in 54 premature infants in two neonatal intensive care units than in the general population. For 42 of these infants, we evaluated urinary concentrations of several phenols, including bisphenol A (BPA), in association with the use of the same medical devices.

**Measurements:**

We measured the urinary concentrations of free and total (free plus conjugated) species of BPA, triclosan, benzophenone-3, methyl paraben, and propyl paraben.

**Results:**

The percentage of BPA present as its conjugated species was > 90% in more than three-quarters of the premature infants. Intensity of use of products containing DEHP was strongly associated with BPA total concentrations but not with any other phenol. Adjusting for institution and sex, BPA total concentrations among infants in the group of high use of DEHP-containing products were 8.75 times as high as among infants in the low use group (*p* < 0.0001). Similarly, after adjusting for sex and DEHP-containing product use category, BPA total concentrations among infants in Institution A were 16.6 times as high as those among infants in Institution B (*p* < 0.0001).

**Conclusion:**

BPA geometric mean urinary concentration (30.3 μg/L) among premature infants undergoing intensive therapeutic medical interventions was one order of magnitude higher than that among the general population. Conjugated species were the primary urinary metabolites of BPA, suggesting that premature infants have some capacity to metabolize BPA. The differences in exposure to BPA by intensity of use of DEHP-containing medical products highlight the need for further studies to determine the specific source(s) of exposure to BPA.

Bisphenol A [BPA; 2,2-bis(4-hydroxyphenyl) propane] is a high-production volume chemical used primarily in manufacturing polycarbonate plastic and epoxy resins that can be used in impact-resistant safety equipment, baby bottles, as protective coatings inside metal food containers, and as composites and sealants in dentistry (Chapin et al. 2008; European Union 2003, 2008; Health Canada 2008; National Toxicology Program 2008).

In experimental animals, exposure to BPA at high doses is associated with estrogen-like effects. At doses below the putative lowest observed adverse effect level, exposure to BPA has resulted in increased prostate gland volume, altered development and tissue organization of the mammary gland, changes in mammary and prostate gland development that may predispose to neoplasia, disruption of sexual differentiation in the brain, and accelerated puberty in females (Ceccarelli et al. 2007; Della Seta et al. 2006; Durando et al. 2007; Gioiosa et al. 2007; Ho et al. 2006; Howdeshell et al. 1999; Laviola et al. 2005; Murray et al. 2007; Negishi et al. 2004; Palanza et al. 2002; Ryan and Vandenbergh 2006; Timms et al. 2005). The interpretation of the evidence related to the low-dose effects of BPA is a subject of scientific debate (Chapin et al. 2008; European Union 2003; Goodman et al. 2008; Gray et al. 2004; National Toxicology Program 2008; vom Saal and Hughes 2005).

Widespread exposure to BPA among the general population (Calafat et al. 2008c; Center for the Evaluation of Risks to Human Reproduction 2007; National Toxicology Program 2008; Vandenberg et al. 2007) likely results from ingesting food containing BPA (Kang et al. 2006; Vandenberg et al. 2007). Acute exposure to BPA may occur in occupational settings and during treatment with dental materials containing BPA (Chapin et al. 2008; European Union 2003; Vandenberg et al. 2007). BPA may also be used in the thermal paper and polyvinyl chloride (PVC) industries (European Union 2003, 2008; National Toxicology Program 2008). In turn, PVC is used in manufacturing medical products, including those found in neonatal intensive care units (NICUs), such as bags containing intravenous fluids and total parenteral nutrition and tubing associated with their administration; nasogastric and enteral feeding tubes; respiratory masks and endotracheal tubes; and umbilical catheters. Di(2-ethylhexyl) phthalate (DEHP) is a plasticizer added to PVC in a variety of these medical products (Food and Drug Administration 2001). Four additional phenols, namely benzophenone-3 (BP-3), methyl paraben (MePB), propyl paraben (PrPB), and triclosan (TCS), may also have potential applications in health- and in personal care scenarios. TCS is a broad-spectrum antimicrobicide used extensively in medical settings (Jones et al. 2000; Weber and Rutala 2006). BP-3 is used as a sunscreen agent in numerous cosmetics and everyday products (Gonzalez et al. 2006). MePB and PrPB are antimicrobial preservatives used in personal care products and pharmaceuticals (Cashman and Warshaw 2005). TCS, BP-3, MePB and PrPB may be endocrine disruptors (Darbre and Harvey 2008; Schlumpf et al. 2001; U.S. Environmental Protection Agency 2008), and human exposure to these compounds is widespread in the United States (Calafat et al. 2008a, 2008b; Ye et al. 2006a). However, the potential health effects of these compounds in humans are largely unknown.

Except for DEHP and other phthalates, limited data exist on the potential exposure to environmental chemicals, including BPA and the other four phenols, in premature infants undergoing intensive medical treatments. Early-life exposures are of great concern with regard to the potential for adverse health consequences throughout the life span. Because premature infants in intensive care units are both developmentally and physiologically immature, they are a potential high-risk population following exposure to environmental chemicals. We demonstrated previously that the intensity of use of products containing DEHP in 54 NICU premature infants is associated with exposure to DEHP, as reflected in urinary concentrations of DEHP metabolites that were substantially higher than those among the general population (Green et al. 2005; Weuve et al. 2006). In this follow-up report, we studied these premature infants’ intensity of exposure to DEHP-containing medical devices in relation to urinary concentrations of several environmental phenols, including BPA.

## Methods

### Study population

In 2003, we studied a convenience sample of 54 low-birth-weight infants from level III NICUs (which provide all newborn care, including mechanical and high-frequency ventilation, surgery, and cardiac catheterization) at two major Boston-area (Massachusetts) hospitals (institutions A and B), as previously described in detail (Green et al. 2005). We chose these infants to reflect a range of diagnoses (including congenital anomalies and developmental and metabolic abnormalities) and NICU care requirements (e.g., ventilation, enteral feedings, parenteral nutrition, indwelling catheterization). The eligibility criteria included *a*) being a patient in the NICU at least 3 consecutive days before enrollment; *b*) having a corrected gestational age (gestational age at birth plus age after birth) of ≤ 44 weeks; and *c*) having been born at or transferred to either hospital between 1 March and 30 April 2003. We excluded infants with hyperbilirubinemia (> 20 μg/dL), which indicates impaired hepatic enzyme function or structural integrity (e.g., biliary atresia). We determined by observation (we had no access to medical records) length of stay in the NICU, primary diagnosis, exposure to medical products, gestational age, sex, and whether the infant was breast- or formula-fed. We also collected at least one urine sample per infant as normally discarded human waste; a protocol-derived record number linked each urine sample to an infant. We had no access to any personal identifiable information, and therefore did not conduct parental interviews or seek parental consent. The original study protocol was approved by the Institutional Review Boards (IRB) of Harvard School of Public Health, Brigham and Women’s Hospital, and Massachusetts General Hospital. In 2008, a protocol for the use of the urine samples and data collected using the original protocol for assessing exposure to BPA and other phenols was deemed exempt from IRB review at Harvard School of Public Health according to 45 Code of Federal Regulations 46.102(d) and/or (f) (Department of Health and Human Services 2005).

### Intensity of use of products containing DEHP

Before data collection in 2003, we defined three levels of intensity of use of products containing DEHP (low, medium, and high) based on a review of medical products typically used in both NICUs and manufacturer-provided information on the products’ DEHP content. The low-DEHP category included infants receiving primarily bottle and/or gavage feedings. The medium-DEHP group included infants receiving enteral feedings by indwelling gavage tubes either continuously or by bolus feedings; intravenous hyperalimentation by indwelling percutaneous intravenous central catheter line or broviac or umbilical vessel catheter; and/or continuous positive airway pressure by nasal prongs. The high-DEHP category included infants receiving continuous indwelling umbilical vein catheterization, endotracheal intubation, intravenous hyperalimentation, and having an indwelling gavage tube (for gastric decompression). As previously described in detail (Green et al. 2005), one of the study investigators observed the care of each infant and, during this observation period, inventoried the products used to care for each infant. Given this inventory, *a priori* we grouped infants into the categories of low-, medium-, and high-use of DEHP-containing products. Product use groups did not change for any of the infants over the course of observation. As reported previously, the intensity of DEHP-containing product use was strongly related to the urinary concentrations of DEHP metabolites (Green et al. 2005; Weuve et al. 2006). For example, among the infants included in the present study, median urinary concentrations of mono(2-ethylhexyl) phthalate, one of the DEHP metabolites, were 6.5, 27, and 92 μg/L, respectively, for those in the low-, medium- and high-DEHP-containing product use groups.

### Urine collection and analysis

As described before (Green et al. 2005), in 2003, spot urine samples were collected at the end of each infant’s observation period from a cotton gauze placed in the infant’s diaper or from the cotton filling of the diaper (we assumed a 100% recovery of free and conjugated urinary species from the diaper or cotton gauze for all compounds examined). In 2003, these urine samples were analyzed for phthalate metabolites at the Centers for Disease Control and Prevention (Atlanta, GA) and the remaining samples were stored under controlled conditions at −40°C. In 2008, for this secondary analysis, we measured BPA concentrations in 57 urine archived samples collected from 41 infants, for some of whom we had up to four samples. Of the 16 replicates, four were collected concurrently with the first samples (i.e., the diaper/cotton gauze yielded enough urine for more than one vial), and the remaining samples were collected between 6 and 48 hr after the first samples. In addition, we measured BP-3, PrPB, and TCS in 59 samples (from 42 infants), and MePB in 58 samples (from 41 infants). At subfreezing storage temperatures, the conjugated species of the phenols examined are known to be stable for 6 months (Ye et al. 2007), and unpublished data from our laboratory extends this stability to at least 30 months. Under the controlled sample storage conditions of this study, we assumed that both free and total (free plus conjugated) species remained stable. Nonetheless, the estimated urinary concentrations of the free species must be interpreted with caution because at the time of the collection of the specimens, we did not pre-screen the sampling materials for the presence of these phenols.

We measured the free and total urinary concentrations of BPA, TCS, BP-3, MePB, and PrPB using online solid-phase extraction coupled to high-performance liquid chromatography (HPLC)–isotope dilution tandem mass spectrometry with peak focusing as described before (Ye et al. 2005, 2006b). Briefly, the conjugated species of the phenols in 100 μL of urine were hydrolyzed with β-glucuronidase/sulfatase (*Helix pomatia*); this deconjugation step was omitted when measuring the concentrations of the free species. After hydrolysis, samples were acidified with 0.1 M formic acid; the phenols were preconcentrated by online solid phase extraction, separated by reversed-phase HPLC, and detected by atmospheric pressure chemical ionization–tandem mass spectrometry. The limits of detection (LODs)—calculated as 3S_0_, where S_0_ is the standard deviation as the concentration approaches zero (Taylor 1987)—were 0.2 μg/L (PrPB), 0.4 μg/L (BPA, BP-3), 1.0 μg/L (MePB), and 2.3 μg/L (TCS). We analyzed low-concentration (~4 to ~25 μg/L) and high-concentration (~10 to ~65 μg/L) quality-control materials, prepared with pooled human urine spiked with the analytes of interest, with standard, reagent blank, and infants’ samples. Samples with concentrations of the analytes above the highest calibration standard were re-extracted using less urine, and the concentrations were calculated after adjusting for the dilution.

### Assessment of other variables

Because the urine sample collection was anonymous and not based on review of medical records, information on gestational age, length of stay in the NICU, and whether the infant was breast- or formula-fed was not available or was incomplete for many infants. We used the intensity of use of products containing DEHP, which correlates with the degree of medical support required, as a blunt surrogate measure for illness severity (Weuve et al. 2006). Nonetheless, because of the anonymous design of the study, we were unable to distinguish levels of illness severity within product-use group. We were able to observe breast- and formula-feeding status of 24 of the infants. Eleven infants were breast-fed (or fed breast milk), of whom 3 were also receiving formula. In contrast, 16 infants were formula-fed (including the 3 who were also breast-fed). We had BPA measurements for 14 of the formula-fed infants, but only for 4 of the breast-fed infants.

### Statistical analysis

Unless noted, we performed the statistical analyses using the total concentrations of the phenols. For each phenol, urinary concentrations below the LOD were assigned a value of LOD divided by the square root of 2 (Hornung and Reed 1990). We computed Spearman correlations between the free and total concentrations of BPA and, to evaluate the variability of urinary concentrations in specimens collected from the same infant, between replicate measures. In addition, to explore the co-occurrence of exposures to phenols and phthalates in the NICU environment, we computed Spearman correlations among urinary concentrations of the phenols and the five phthalate metabolites we evaluated in our previous work (Green et al. 2005; Weuve et al. 2006). For these analyses we computed both crude and institution-adjusted correlations, because institution confounded the associations between BPA and the DEHP metabolites. For the crude comparisons of urinary concentrations of each phenol by sex, institution, or DEHP-containing product-use group, we used the Kruskal–Wallis nonparametric test. We also used linear mixed effects regression to compare urinary concentrations of each phenol across DEHP-containing product-use groups, adjusting for institution and infants’ sex, and accounting for the correlations between replicate measures among the same infants. We fit separate regression models for each phenol, using the first two concentrations available for each infant, resulting in 54, 56, 56, and 55 urine samples used in the models of BPA, BP-3, PrPb, and MePB, respectively. We did not fit models for TCS because too few urine samples had detectable levels of this phenol. To stabilize the variances of the urinary phenol concentrations, we used the log-transformed concentration as the dependent variable in the regression models. Therefore, when exponentiated, the estimated regression parameters in each phenol model are interpreted as the urinary concentration for a given independent variable expressed as a proportion (or multiple) of the urinary concentration in that variable’s reference level; for example, in the BPA model, the exponentiated regression parameter for the medium-DEHP product-use group is the estimated urinary BPA concentration in this group as a proportion of that in the low-DEHP product-use group. We conducted all analyses with SAS software, version 9.1.3 (SAS Institute Inc., Cary, NC).

## Results

**Figure 1:**
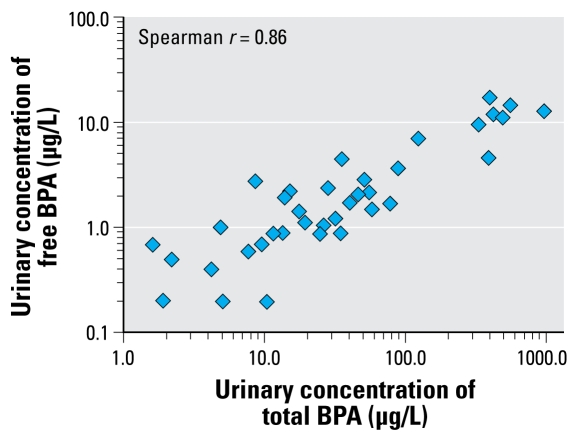
Correlation between free and total BPA urinary concentrations for 37 premature infants, displayed in log-10 scale (*r* = 0.86).

Forty-one premature infants had at least one urine sample available for analysis of BPA and MePB (42 for TCS, BP-3, and PrPB). We detected BPA in all of the first set of urine samples collected from these infants at total concentrations ranging from 1.6 to 946 μg/L; the geometric mean was 30.3 μg/L (Table 1). MePB and PrPB were also detected in all of the samples and BP-3 in all but two samples (Table 1). TCS was detected in approximately 19% of the samples and therefore was not included in any further statistical analysis. Of interest, the free species of some of these phenols were detected slightly less frequently and at substantially lower concentrations than the conjugated species (Table 1). For example, free BPA was detected in 92% of the samples; the median (1.7 μg/L) and highest concentrations (17.3 μg/L) were an order of magnitude lower than the total concentrations. Of interest, the concentrations of free and conjugated species of BPA showed a linear relationship (Spearman correlation, *r* = 0.86) throughout the range of total BPA concentrations observed (Figure 1). Because of the limited amount of urine available for analysis, the concentration of free species could not be measured in all samples.

The infants in this study were roughly equally distributed between the two institutions, and about 38% (information on sex was missing for one of the infants) were male (Table 2). Fewer infants were exposed to DEHP-containing products at low intensity of use than at medium or high intensity of use (Table 2).

Two or more replicate samples were available for 14 infants (13 for BPA); we used the first two of these samples to assess variability in urinary concentrations within individual infants (Table 3). The repeated urinary measurements from the same infants were highly correlated for BPA and BP-3, and also correlated, although to a lesser extent, for MePB and PrPB. As expected, the correlations between the concurrent (i.e., more than one urine sample collected from a single diaper or cotton gauze) measurements were excellent (all Spearman *r* ≥ 0.95) (Table 3).

As has been reported (Ye et al. 2006a), we found that institution-adjusted urinary concentrations of MePB and PrPB were highly correlated (Spearman *r* = 0.73, *p* < 0.0001), suggesting that human exposures to these para-bens most likely share common pathways. BPA was moderately correlated with MePB (*r* = 0.40, *p* = 0.01). Notably, however, BPA was correlated with the phthalate metabolites, including the three metabolites of DEHP among which it was strongly correlated with DEHP oxidative metabolites mono(2-ethyl-5-hydroxy-hexyl phthalate (*r* = 0.57, *p* < 0.0001) and mono(2-ethyl-5-oxohexyl) phthalate (*r* = 0.57, *p* < 0.0002). The remaining institution-adjusted correlations among the different phenols with each other and with the phthalate metabolites measured before (Green et al. 2005; Weuve et al. 2006) were weak to moderate (Table 4).

Of the 41 infants who had at least one BPA measure, gestational age data were available for only 16 of them. After dividing the group roughly along the median gestational age (25–27 weeks vs. 28–34 weeks), the median total BPA concentrations was higher (Kruskal–Wallis *p*-value = 0.06) for the younger (242 μg/L) than for the older (29 μg/L) infants (based on gestational age). For the 25 infants for whom we had information about length of stay in the NICU, BPA concentrations did not appear to vary across long (14–90 days) versus short (2–13 days) length of stay. The difference in BPA concentrations for the breast-fed (or fed breast milk) infants (*n* = 4) and the formula fed infants (*n* = 14) was not statistically significant (*p* = 0.7).

In the crude analyses, urinary BPA concentrations were significantly higher among infants at institution A than at institution B (*p* < 0.0001; Table 2). Concentrations of the other phenols measured were similar across institutions (Table 2). Urinary concentrations of BPA and the other three phenols did not vary substantially by infants’ sex, and no consistent associations were present between intensity of DEHP-containing product use and urinary concentrations of any of the phenols examined (Table 2).

On adjusting for infants’ sex and intensity of use of products containing DEHP, the association between institution and BPA concentrations persisted (Table 5). On average, urinary concentrations of BPA among infants hospitalized at institution A were almost 17 times greater than concentrations among infants at institution B (*p* < 0.0001). By contrast, infants hospitalized at Institution A had BP-3 urinary concentrations that were, on average, about one-third the concentrations among infants at institution B (*p* = 0.04). Concentrations of the two parabens did not vary consistently by DEHP-containing product use group or institution. In these adjusted analyses, none of the phenol concentrations varied by infants’ sex.

The institution- and sex-adjusted associations between the use of medical products containing DEHP group and urinary concentrations were highly significant for BPA but not for any of the other phenols (Table 5). Compared with infants exposed at low intensity to DEHP-containing products, infants exposed at medium intensity had BPA concentrations that were 3.42 times as high [95% confidence interval (CI), 1.45–8.09], and infants exposed at high intensity had concentrations that were 8.75 times as high (95% CI, 3.36–22.8). The results of this sex- and institution-adjusted analysis differed from the crude analyses, shown in Table 2, which did not show marked differences in BPA concentration by DEHP group. Similarly, results from linear mixed effects regression models of BPA that did not adjust for sex or institution also showed no significant differences in BPA concentration by DEHP-containing product use group (data not shown). These different findings were most likely attributable to negative confounding by institution; infants at institution B had lower BPA concentrations but were much more likely than infants at institution A to be exposed at high intensity (29% vs. 7%) to DEHP-containing products.

## Discussion

We frequently detected BPA and three other phenols (BP-3, MePB, and PrPB) in this group of premature infants. The median urinary concentrations of BP-3 in these infants were about one order of magnitude lower than the concentrations reported by the National Health and Nutrition Examination Survey (NHANES) 2003–2004 for children 6–11 years of age in the U.S. general population (Calafat et al. 2008a). Similarly, the low frequency of detection of TCS among these infants suggests that, although TCS may be in products used by workers in health care settings, infants’ exposure to TCS in NICUs does not appear to be higher than that of the general population. We found that the median urinary concentrations of MePB and PrPB in the infants examined were higher than the median observed among a group of 100 adults in the United States (Ye et al. 2006a), but lower than the 95th percentile concentrations in the same group of adults; no data exist on the extent of paraben exposure among children. These results suggest that the use of products that contain parabens in the treatment of NICU premature infants can result in exposure levels that may be higher than those observed among the general population.

By contrast, we found that the median urinary concentrations of BPA among the infants (28.6 μg/L) were about one order of magnitude higher than the median concentration (3.7 μg/L) and almost twice the 95th percentile concentration (16.0 μg/L) among children 6–11 years of age who were examined as part of the NHANES 2003–2004 (Calafat et al. 2008c). These data suggest that exposure to BPA among the infants in our study was much higher than among any of the populations examined in the United States (Chapin et al. 2008; National Toxicology Program 2008). Furthermore, that > 90% of the BPA excreted in the urine was in its conjugated (e.g., glucuronide, sulfate) form excludes the possibility that the BPA concentrations measured in these infants’ urine resulted primarily from contamination and supports the validity of our analytical data. More important, our findings suggest that even premature infants have some capacity to conjugate BPA, in agreement with previous studies suggesting that critically ill premature infants could, to a certain extent, metabolize DEHP metabolites to their urinary glucuronides (Calafat et al. 2004). Furthermore, the fact that the association between the concentrations of free and total BPA was linear across the range of observed total BPA concentrations suggests that saturation of the enzyme(s) responsible for the conjugation of BPA did not occur even at total BPA concentrations orders of magnitude higher those reported among the general U.S. population. Nevertheless, although shortly after birth some hepatic enzymes involved in phase II metabolism (such as UDP-glucuronosyltransferases) can be activated even in premature infants (Blake et al. 2005; Burchell et al. 1989; Jansen et al. 1992; Leakey et al. 1987), these glucuronidation pathways are not expected to be functional at adult rates until months after birth (de Wildt et al. 1999). Studies designed to confirm these preliminary findings are warranted to examine the percentage of free BPA and conjugation ability of infants and young children, including premature infants.

In the crude analyses, the median total BPA concentrations were about one order of magnitude higher among the infants 25–27 weeks of age than among the older (28–34 weeks) infants. Of interest, among the younger infants (gestational age, 25–27 weeks), five were in the medium-intensity and three were in the high-intensity group exposed to DEHP-containing products. By contrast, five of the older infants (gestational age, 28–34 weeks) were in the low-intensity and three were in the medium-intensity group. Therefore, the observed differences in BPA concentrations by gestational age are likely to be attributable partially to the treatment given to the infants. On the other hand, the length of stay in the NICU or feeding method (formula vs. breast milk) did not result in significant differences in BPA concentrations. However, because information on gestational age, length of stay in the NICU, and feeding type was incomplete for many infants, these results are preliminary; the potential association of these variables with BPA exposure should be addressed in future research.

In this follow-up study of premature infants receiving care in two NICUs, we found moderate to strong institution-adjusted correlations between urinary concentrations of BPA and the metabolites of DEHP, indicating that BPA and DEHP may share common exposure sources. Consistent with this possibility, we found strong associations between the use of DEHP-containing medical devices and urinary concentrations of BPA. Specifically, after adjustment for infants’ sex and institution, urinary BPA concentrations among infants who were exposed at high intensity to DEHP-containing products were almost nine times as high as those in the low-intensity group. Urinary BPA concentrations among infants who were exposed at medium intensity to DEHP-containing products were more than three times as high as those in the low-intensity group. Furthermore, urinary BPA concentrations were higher among infants hospitalized at institution A than among infants hospitalized at institution B, regardless of DEHP product-use group. Of interest, the urinary concentrations of DEHP metabolites in infants of institution B were higher than in infants of institution A and might have reflected the extensive use of two DEHP-containing PVC medical devices at institution B that were used sparingly in institution A (Green et al. 2005; Weuve et al. 2006). We have no information about the presence of BPA in any of the medical or other products used in the care of the infants. Therefore, we cannot rule out that the two institutions used products for the care of the infants that also differed in their BPA content which may have contributed to the observed differences.

Moreover, BPA may leach from other baby and consumer products, including those made from polycarbonate plastic and epoxy resins (European Union 2008; Health Canada 2008; National Toxicology Program 2008); unfortunately, we did not collect information related to the use of these products. Taken together, these findings suggest that exposure to BPA in NICUs may result from the use of specific medical products; these products may also be sources of exposure to other compounds, such as DEHP. However, this study’s small size, narrow range of descriptive data on the infants and the products used for their care (except for those related to DEHP content), and lack of environmental measures from the infants’ NICU surroundings limited our ability to conduct more detailed analyses.

Although the data suggest the presence of BPA in medical devices/products, we can not determine with certainty the source(s) of BPA because we did not test for BPA in the medical devices and products used or the dietary liquids ingested (e.g., breast milk or formula). Therefore, we cannot rule out that the milk or formula the infants consumed was the source of BPA exposure, as BPA has been detected both in human milk and formula (National Toxicology Program 2008). However, we have no reason to suspect that the amounts of milk or formula consumed by the premature infants differed considerably by the use of medical products containing DEHP and the institution in which they were patients.

To our knowledge, this study is the first to describe infants’ exposure to BPA, TCS, BP-3, MePB, and PrPB. In conjunction with the previous data collected on phthalates for the same group of premature infants (Green et al. 2005; Weuve et al. 2006), our findings suggest that infants may be exposed during critical periods of their development to several potential reproductive and developmental toxicants at levels higher than those reported for the general population. However, this was an exposure study, and we were therefore unable to explore whether such exposures were associated with adverse health effects in these infants. This study is also the first to demonstrate that increasing intensity of DEHP-containing PVC product use is proportional to exposure to BPA, as was reflected in significant elevations in urinary concentrations of BPA and the absence of similar elevations in urinary concentrations of the other four additional phenols examined. Concerns related to BPA toxicity as well as high BPA exposure levels in this sensitive population of low-birth-weight premature infants may justify using products that do not contain BPA while not compromising the quality of medical care. Future research is needed to establish the source(s) and the potential health outcomes of these exposures to BPA.

## Figures and Tables

**Table 1:** Distribution of the urinary concentrations of phenols (μg/L)^a^ in hospitalized premature infants.

Compound	Species	No. of infants	No. < LOD	Geometric mean (SD)	Median	Range		NHANES 2003–2004^b^	
						Minimum	Maximum	Median	95th percentile
BP-3	Total	42	2	3.4 (4.8)	2.4	< LOD (0.4)	176	17.2	227
	Free	36	17	NA	NA	< LOD (0.4)	4.1		

BPA	Total	41	0	30.3 (5.2)	28.6	1.6	946	3.7	16.0
	Free	37	3	1.8 (3.2)	1.7	< LOD (0.4)	17.3		

MePB	Total	41	0	203 (4.7)	243	10.1	4,010	43.9^c^	680^c^
	Free	34	0	32 (4.9)	23	2.2	515		

PrPB	Total	42	0	16.8 (4.9)	17.0	1.3	1,360	9.1^c^	279^c^
	Free	37	0	2.6 (5.2)	1.7	0.3	171		

TCS	Total	42	34	NA	NA	< LOD (2.3)	16.7	5.9	148
	Free	37	36	NA	NA	< LOD (2.3)	3.4		

a The total concentrations are the sum of the free plus conjugated species of each phenol. We calculated geometric means and medians if the frequency of detection was > 60%. The estimated concentrations of free species must be interpreted with caution because at the time of the collection of the urine specimens, we did not prescreen the sampling materials for the presence of these phenols.

b Data from 314 children 6–11 years of age from NHANES (National Health and Nutrition Examination Survey) 2003–2004 for BP-3 (Calafat et al. 2008a), BPA (Calafat et al. 2008c), and TCS (Calafat et al. 2008b).

c Data from a group of 100 dults (Ye et al. 206).

**Table 2:** Median and 25th and 75th percentile concentrations of urinary phenols (μg/L) in hospitalized premature infants, by intensity of use of products containing DEHP, institution, and sex.

	BP-3					BPA					MePB					PrPB				
	No.	25th	Median	75th	*p*-Value^a^	No.	25th	Median	75th	*p*-Value^a^	No.	25th	Median	75th	*p*-Value^a^	No.	25th	Median	75th	*p*-Value^a^
DEHP-containing product use																				
High	15	1.2	2.6	7.6	0.5	14	10.4	24.0	54.6	0.9	15	158	340	1,450	0.07	15	11.7	21.3	44.5	0.3
Medium	17	1.1	1.3	4.4		17	4.8	46.7	88.7		17	43	114	325		17	3.4	8.9	19.1	
Low	10	1.6	4.1	7.6		10	11.5	29.6	35.6		9	69	177	1,560		10	2.9	24.9	142	

Sex																				
Male	16	1.0	3.3	10.8		16	9.1	26.9	210.9		16	103	333	553		16	6.3	15.1	53.9	
Female	25	1.2	1.9	3.6	0.7	24	8.3	27.1	54.8	0.6	24	52	182	534	0.3	25	5.3	18.3	44.3	1

Institution																				
A	20	1.0	1.8	5.4	0.2	20	29.6	69.9	391	< 0.0001	19	32	317	598	0.8	20	2.8	7.1	40.0	0.1
B	22	1.2	2.9	12.8		21	4.9	9.5	28.6		22	63	191	496		22	12.0	18.7	61.2	

a Kruskal–Wallis nonparametric test for differences by group.

**Table 3:** Spearman correlations between repeated urinary concentrations among premature infants (first two specimens).

	No. of infants with ≥ 2 measurements	Spearman correlation	*p*-Value	No. of concurrent measurements	Spearman correlation	*p*-Value
BP-3	14	0.86	< 0.0001	4	0.95	0.05
BPA	13	0.97	< 0.0001	4	1.00	< 0.0001
MePB	14	0.62	0.02	4	1.00	< 0.0001
PrPB	14	0.47	0.09	4	1.00	< 0.0001

**Table 4:** Institution-adjusted Spearman correlations between the total urinary concentrations of different analytes among premature infants (all *n* = 41 or 42).

	BPA	MePB	PrPB	MEHP^a^	MEHHP^a^	MEOHP^a^	MBP^a^	MBzP^a^
BP-3	−0.04	0.16	0.02	−0.10	−0.08	−0.06	0.11	0.06
*p*-Value	0.8	0.3	0.9	0.6	0.6	0.7	0.5	0.7

BPA		0.40	0.21	0.29	0.57	0.57	0.31	0.32
*p*-Value		0.01	0.2	0.08	0.0001	0.0002	0.06	0.05

MePB			0.73	0.29	0.34	0.43	0.28	0.12
*p*-Value			< .0001	0.08	0.03	0.01	0.09	0.5

PrPB				0.17	−0.01	0.07	−0.06	−0.08
*p*-Value				0.3	0.9	0.7	0.7	0.6

Abbreviations: MBP, monobutyl phthalate; MBzP, monobenzyl phthalate; MEHHP, mono(2-ethyl-5-hydroxyhexyl) phthalate; MEHP, mono(2-ethylhexyl) phthalate; MEOHP, mono(2-ethyl-5-oxohexyl) phthalate.

a Data from Green et al. (2005); Weuve et al. (2006).

**Table 5:** Multivariable-adjusted^a^ relative urinary concentrations of the phenols^b^ (95% CI) in hospitalized premature infants, by use of DEHP-containing products, infant’s sex, and institution.

	BP-3 (*n* = 56)		BPA (*n* = 54)		MePB (*n* = 55)		PrPB (*n* = 56)	
	Concentration (95% CI)	*p*-Value	Concentration (95% CI)	*p*-Value	Concentration (95% CI)	*p*-Value	Concentration (95% CI)	*p*-Value
DEHP-containing product use								
High	0.36 (0.08–1.53)		8.75 (3.36–22.8)		2.48 (0.76–8.05)		1.09 (0.30–3.93)	
Medium	0.52 (0.14–1.93)	0.36^c^	3.42 (1.45–8.09)	0.0003^c^	0.65 (0.21–1.99)	0.01^c^	0.59 (0.18–1.96)	0.4^c^
Low	1 (Referent)		1 (Referent)		1 (Referent)		1 (Referent)	

Sex								
Male	1.61 (0.57–4.49)	0.4^c^	1.07 (0.55–2.08)	0.9^c^	1.53 (0.68–3.46)	0.3^c^	0.96 (0.39–2.36)	0.9^c^
Female	1 (Referent)		1 (Referent)		1 (Referent)		1 (Referent)	

Institution								
A	0.31 (0.10–0.96)	0.04^c^	16.6 (7.98–34.6)	< 0.0001^c^	1.29 (0.54–3.08)	0.6^c^	0.44 (0.17–1.16)	0.1^c^
B	1 (Referent)		1 (Referent)		1 (Referent)		1 (Referent)	

a Each metabolite is represented by its own model that is adjusted for DEHP-containing-product use group, sex, and institution.

b Results expressed as multiples of the concentrations in the reference group. For example, infants in the high use of DEHP-containing products group have BPA concentrations nearly 9 times the concentrations among infants in the low use of DEHP-containing product group.

c *p*-Value corresponds to overall association between variable and phenol concentration.
